# A stem cell journey in ophthalmology: From the bench to the clinic

**DOI:** 10.1002/sctm.21-0239

**Published:** 2021-09-13

**Authors:** Ingrid W. Caras, Lila R. Collins, Abla A. Creasey

**Affiliations:** ^1^ The California Institute for Regenerative Medicine Oakland California USA

**Keywords:** cellular therapy, clinical translation, retina, retinal pigmented epithelium, stem cells

## Abstract

Debilitating diseases of the eye represent a large unmet medical need potentially addressable with stem cell‐based approaches. Over the past decade, the California Institute for Regenerative Medicine (CIRM) has funded and supported the translation, from early research concepts to human trials, of therapeutic stem cell approaches for dry age‐related macular degeneration, retinitis pigmentosa, and limbal stem cell deficiency. This article chronicles CIRM's journey in the ophthalmology field and discusses some key challenges and questions that were addressed along the way as well as questions that remain.

## Graphical abstract

Cell‐based therapies comprised of or derived from stem/progenitor cells are being developed to treat debilitating eye diseases such as dry age‐related macular degeneration (AMD), retinitis pigmentosa (RP), and limbal stem cell deficiency (LSD). Shown are the routes of delivery and location within the eye of the specific cell therapies discussed in this article. Adapted from https://www.freepik.com/premium-vector/eye-anatomy-vector_760161.htm.

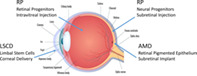

**Significance statement**

Debilitating eye diseases represent a large unmet medical need potentially addressable by stem cell‐based approaches. This article describes the advances made over the past 10 years by California Institute for Regenerative Medicine‐supported grantees in developing and translating stem cell‐based therapies for dry age‐related macular degeneration, retinitis pigmentosa, and limbal stem cell deficiency. The approaches described are now under evaluation in the clinic or have obtained approval to begin a clinical trial.

## 1 INTRODUCTION

Loss of vision caused by a debilitating eye disease has a major impact on an individual's mobility, independence, quality of life (QOL), and ability to function in the modern world.

Eye diseases that cause progressive blindness include both age‐related and inherited retinal diseases as well as corneal diseases. Age‐related macular degeneration (AMD) is a leading cause of vision loss in people over 65 years and is currently estimated to affect 11 million Americans and 170 million individuals worldwide (MedlinePlus; https://medlineplus.gov/genetics/condition/age‐related‐macular‐degeneration/#frequency). Although a number of antivascular endothelial growth factor therapies are available for the wet, exudative form of AMD, there are currently no approved therapies to treat the more common dry form (80%‐90% of cases) which destroys central vision needed for reading, driving, and recognizing faces, resulting in significant impact on QOL. Retinitis pigmentosa (RP) is the leading cause of inherited vision loss leading to blindness, affecting approximately 1/4000 individuals (https://medlineplus.gov/genetics/condition/retinitis-pigmentosa/#frequency). It is estimated that 1.5 million people worldwide are currently impacted and there are no approved therapies. RPE65‐mediated retinal dystrophy (Leber's congenital amaurosis) is another rare inherited disorder causing progressive blindness. In 2017, the Food and Drug Administration (FDA) approved Luxturna (voretigene neparvovec), a gene therapy treatment for patients with confirmed biallelic RPE65 mutation‐associated retinal dystrophy. Luxturna is the first approved gene therapy for an inherited retinal disease and the first adeno‐associated virus vector gene therapy approved in the United States. Limbal stem cell deficiency (LSCD) is a rare, progressive corneal disease that is found throughout the world, has wide‐ranging etiology, and can culminate in blindness. Surgical tissue transplantation is the main treatment option, but is often unsuccessful.

Developing treatments for debilitating and blinding eye diseases was a relatively early focus of regenerative medicine research. The accessibility and small size of the eye are an advantage for cell therapy manufacture and delivery. These distinguishing features, combined with the many sophisticated ophthalmic imaging tools for assessing and monitoring clinical outcomes, make the eye an attractive target for evaluating stem cell‐based therapies. Beginning in 2008, the California Institute for Regenerative Medicine (CIRM), together with its grantees, pioneered the development of cell‐based approaches for severe eye diseases including diseases of the retina such as dry AMD and RP as well as the corneal disease, LSCD. This article briefly describes the progress made from the bench to early clinical trials, beginning in the trail blazing years of the now burgeoning field of regenerative medicine. We highlight initial questions and what has been learned along the way as well as remaining questions and potential challenges as these programs progress in clinical development.

## 2 AGE‐RELATED MACULAR DEGENERATION

The first CIRM‐funded stem cell translational awards were launched in 2009 and 2010 and several focused on dry AMD, considered an ideal indication for pioneering a cell replacement approach. Briefly, dry AMD is a slowly progressing disease of the retina, driven by degeneration of the retinal pigment epithelium (RPE), a polarized monolayer of cells that underlies the photoreceptors (PRs) and is essential for maintaining PR viability and function. Loss of RPE leads to PR degeneration and eventual blindness. Early researchers reasoned that if damaged RPE could be replaced with healthy, human embryonic stem cell (hESC)‐derived RPE, disease progression could be arrested or possibly even reversed.

But first, several important questions had to be addressed: Are hESC‐derived RPE cells functionally equivalent to natural RPE cells? Do different hESC lines differ in their capacity to differentiate into RPE and which line should be used to make transplantable RPE for use in humans? Can induced pluripotent stem cells (iPSCs) be used to make viable RPE? Early CIRM grantees addressed these questions and successfully derived transplantable RPE cells from a number of stem cell sources including various hESC and iPSC lines. These teams demonstrated that stem cell‐derived RPE is phenotypically and functionally comparable to primary human RPE based on morphology, gene and protein expression, metabolome, and rod outer segment phagocytosis, a key function of the RPE. They also showed that viable RPE can be derived using iPSC, suggesting that autologous approaches for treating AMD may be feasible.^1^, ^2^, ^3^

This early research relied on the spontaneous differentiation of hESC into RPE when cultured, a process that is slow and yields a low percentage of RPE.^4^ The observation that some hESC lines exhibited more robust RPE differentiation capacity than others, producing higher frequencies of pigmented RPE cells, highlighted the importance of selecting an optimal, well characterized starting cell line before beginning translational activities.

A key question was how to deliver stem cell‐derived RPE to the retina. What devices or tools could be used? Would the cells survive and persist? For RPE to perform their multiple essential functions, which include interacting with and supporting the PR and participating in the visual cycle,^5^ investigators reasoned that the transplanted RPE cells would need to mimic endogenous RPE and form a polarized monolayer in contact with both the underlying Bruch's membrane on which RPE cells depend for survival and the overlying PR that they support. In advanced AMD, known as geographic atrophy (GA), the Bruch's membrane is damaged or dysfunctional, raising concerns about the attachment and survival of transplanted RPE cells and their ability to function in the retina.^6^

Mark Humayun, an early CIRM awardee and cofounder of the California Project to Cure Blindness (CPCB), addressed this concern by designing an implant composed of a polarized monolayer of hESC‐derived RPE (hESC‐RPE) on an ultrathin, synthetic parylene substrate designed to both provide a surface for RPE adhesion and to mimic the diffusion barrier characteristics of Bruch's membrane. In a comparative study in rats, the survival of hESC‐RPE after subretinal implantation was significantly improved when RPE cells were delivered as a polarized monolayer on parylene compared to as a cell suspension.^7^ With CIRM funding, the CPCB developed good manufacturing practice (GMP) procedures to produce transplantable hESC‐derived RPE and manufacture the composite implant. They also developed and validated tools and procedures to surgically insert it into human retinas, completed preclinical safety evaluations and filed an investigational new drug (IND) application for the implant.

A CIRM‐funded phase 1/2a first‐in‐human (FIH) study to test the CPCB hESC‐RPE implant was initiated in 2015 by Regenerative Patch Technologies, a company founded by the CPCB. Surgical delivery was achieved using a specifically designed tool into which the ultrathin composite implant could be folded for insertion through a small incision in the sclera and then positioned over the area of damage in the retina. Data from 16 patients enrolled in the study demonstrated that surgical delivery is feasible in an outpatient setting and that the implant could be successfully targeted to the area of GA.^8^

Follow‐up structural and functional analyses have been reported for five patients in cohort 1 of the study, comprising advanced AMD patients severely affected by GA. These patients had poor visual acuity at baseline and because the area of GA involved the fovea, their ability to visually fixate on an object was unstable or absent. Optical coherence tomography (OCT) imaging suggested that there was integration of the hESC‐RPE with PR in the retinal tissue of the recipient eye. Although patients with advanced GA are thought to have little potential for visual recovery, there were signs of improved visual function. None of the implanted eyes showed further progression of vision loss and one eye showed improvement in visual acuity. Two patients improved their ability to visually fixate, suggesting that the implanted hESC‐RPE monolayer supports visual function in the overlying, previously nonfunctional retina.^9^ The investigators speculate that these improvements may be due to revival of dormant PR in the area of GA, resulting from direct integration with the hESC‐RPE. Importantly, there were no safety concerns. Patients in the study received immunosuppressive therapy for approximately 2 months postimplantation. Analysis of OCT images indicated continued presence of the implanted hESC‐RPE and integration with the recipient retinal tissue. In addition, the observed functional improvements were maintained for at least 120 days at the time of reporting, suggesting persistence of the implanted RPE after immunosuppression was stopped.

### 2.1 Alternative approaches for dry AMD

A number of alternative cell‐therapy approaches are being investigated for dry AMD using different cell types or different starting sources of cells to derive RPE and alternative/no scaffold to deliver the cells (Table 1). All of these approaches are in early stages of clinical development. All but one use allogeneic cells while one approach employs autologous iPSC‐derived RPE. It remains to be seen whether an autologous approach can adequately support a disease with a large patient population such as AMD and whether the potential advantages of an autologous approach (reduced/no immunogenicity) will outweigh the challenges and cost of manufacturing.

**TABLE 1:** Summary of clinical stage cell therapy approaches for dry age‐related macular degeneration

Sponsor	Product	Cell type	Delivery	Phase
Regenerative Patch Technologies	CPCB‐RPE1	Human embryonic stem cell‐derived RPE cells on a parylene membrane	Subretinal implantation	Phase 2a
NIH	iPSC‐derived RPE/PLGA	Autologous iPSC‐derived RPE on a biodegradable poly lactic‐co‐glycolic acid (PLGA) scaffold	Subretinal transplantation	Phase 1/2a
Astellas	ASP7317 (MA09‐hRPE)	Human embryonic stem cell‐derived RPE cells	Subretinal injection	Phase 1/2a
Lineage Cell Therapeutics	OpRegen	Human pluripotent stem cell‐derived RPE cells	Subretinal injection	Phase 1/2a
Janssen Labs	CNTO‐2476 (palucorcel)	Human umbilical tissue‐derived cells (hUTCs) of mesenchymal origin	Subretinal administration using a microcatheter	Phase 2b
Luxa Biotechnology	RPESC‐RPE‐4W	Allogeneic RPE stem cell (RPESC)‐derived RPE cells (RPESC‐RPE) isolated from the RPE layer of human cadaveric eyes	Transplanted under the macular	Phase1/2a

Abbreviations: iPSC, induced pluripotent stem cell; RPE, retinal pigment epithelium.

## 3 RETINITIS PIGMENTOSA

CIRM began funding development of a stem cell therapy for RP in 2008. Briefly, RP is an inherited degenerative disease of the PRs, typically with earlier onset than AMD. The literature documents that RP can be caused by more than one hundred different mutations in multiple genes, many of them rod‐specific, making it challenging to address with a gene therapy approach. RP is characterized initially by loss of the rod PRs that are located in the peripheral retina and are responsible for low‐light vision. Clinically, this anatomical loss of rod PR manifests as loss of peripheral vision, diminished ability to see in low light conditions and difficulty seeing at night. Subsequently, because the more important cone PR that enable high‐resolution color vision rely upon cone survival factors released by the rods, loss of rods leads to loss of cones, eventually culminating in total blindness. Cell‐replacement of lost PR would require re‐establishing synapses and neural connections, events that are likely hard to accomplish. More feasible treatment strategies, at least in the near term, tend to focus on preserving cone PRs, either directly or by preserving the rods. Such strategies could in principle arrest disease progression, which would have a major impact on patients, particularly in earlier stages of disease. Beginning in 2011, CIRM has supported the translation and development of two novel therapeutic approaches for RP.

### 3.1 Retinal progenitor cells

One approach, pioneered by Henry Klassen, is transplantation of cultured, allogeneic retinal progenitor cells (RPCs) of fetal tissue origin into the eyes of patients with RP with the goal of preserving vision by achieving neuroprotection of PRs, particularly the more essential cones. A major advantage of this neuroprotective approach is that the potential to preserve or restore vision is independent of the specific RP mutation causing this heterogeneous disease. Based on studies using mouse RPC transplanted into retinal dystrophic mice, human RPC (hRPC) were initially thought to be protective via both a trophic mechanism as well as by differentiating into rod PR upon which cone survival depends (https://doi.org/10.1167/iovs.04-0511). Preclinical biodistribution and cell survival studies subsequently suggested that the mechanism of neuroprotection is primarily paracrine.

With CIRM funding, the Klassen team developed a GMP process to expand and manufacture hRPC and demonstrated preclinical safety and efficacy of the resultant hRPC. A critical early question that the team addressed was where in the eye to deliver the RPC. Preclinical experiments in a rat model of retinal degeneration compared subretinal vs intravitreal delivery. Subretinal transplantation of RPC showed PR rescue, but it was restricted to the area of the injection. In contrast, intravitreal injection resulted in PR outer nuclear layer preservation across a greater area of the retina, indicating that RPCs survive in the vitreous and are able to broadly support the host retina from this delivery site (H. Klassen, unpublished data). As an added benefit, it was recognized that intravitreal injection greatly facilitates delivery of the cells and allows for the option of retreatment. Preclinical safety studies in both large and small animal models demonstrated the feasibility and tolerability of intravitreal injection of hRPC as well as the absence of an immune response even following a repeat injection. Based on this work, an IND was filed and cleared by the FDA.

With continued CIRM support, a FIH phase 1/2 trial sponsored by jCyte, a company cofounded by Henry Klassen, was initiated in 2015 to evaluate the safety and potential activity of a single dose of hRPC administered intravitreally in one eye of 28 adults with RP. This study demonstrated that intravitreal injection of hRPC was well tolerated at doses up to 3 million cells (https://iovs.arvojournals.org/article.aspx?articleid=2690954). Although not powered for efficacy, changes in best‐corrected visual acuity (BCVA) in treated eyes were found suggestive of a therapeutic benefit at the higher dose levels.

Based on these encouraging results, a masked, randomized phase 2b study to evaluate the safety and efficacy of a single intravitreal injection of hRPC in adult patients with RP was initiated in 2017. Patients received either 3 million or 6 million cells or sham treatment in one eye. Promising results from this trial were announced in July 2020 at the American Society of Retina Specialists Annual Meeting. Evaluation of all 74 enrolled and evaluable patients at 12 months post‐treatment showed a trend of improvement in BCVA in patients who received the higher cell dose compared to the sham control. Those receiving 6 million cells had a mean improvement in BCVA of 7.43 letters while those receiving 3 million cells or sham had mean BCVA improvements of 2.96 and 2.81 letters, respectively (a standard eye chart used for visual acuity testing has five letters per line). Post hoc analysis of a subset of 37 patients representing a target patient population for a subsequent trial showed a statistically significant improvement in BCVA compared to sham control at the 6 million cell dose level, with corroborating improvements in secondary endpoints including low light mobility, contrast sensitivity, visual fields, and QOL (the latter assessed by a visual function questionnaire). Based on the encouraging positive results from this phase 2b trial, a phase 3 trial is being planned.

### 3.2 Innovative clinical endpoints for RP

For patients with RP, the ability to safely navigate activities of daily living can be severely impacted by the decreasing visual field and loss of light sensitivity despite relative preservation of visual acuity in the narrowing central field. Although BCVA is widely accepted as the gold standard endpoint for measuring visual function, it does not capture all aspects affecting QOL and is thought not to assess loss of peripheral vision or ability to see at night, both of which are impaired in RP patients.

To more broadly evaluate the effects of hRPC therapy in RP patients, the jCyte team developed and validated a novel Low Light Mobility assay for inclusion in the phase 2b trial. This assay measures the lowest light level at which the patient can functionally navigate a maze. A similar multiluminance mobility test that assesses functional vision was used as the primary endpoint of the phase 3 clinical trial of voretigene neparvovec (Luxturna) and was the basis for its approval.^10^

RP investigators continue to tackle a number of questions related to the appropriate evaluation of therapies for this indication. Since patients with RP lose vision in both eyes, should treatment be administered to both eyes simultaneously or sequentially? What is the durability of the clinical effect? In the jCyte phase 2b study, effects were durable for at least 12 months, but it is anticipated that repeat dosing will be required and the final dosing regimen remains to be determined.

### 3.3 Cortical neural progenitor cells

Beginning in 2015, CIRM funded late preclinical development of a complementary allogeneic neuroprotective strategy for RP that uses subretinal injection of CNS10‐NPC, a neural progenitor cell population derived from human fetal cortex. In rodent and non‐human primate models, subretinally delivered CNS10‐NPC migrate beyond the injection site to durably engraft as a layer between the RPE and PRs.^11^, ^12^ Engrafted cells have been shown to persist for at least 9 months in rodents.^13^ Intriguingly, these cells exhibit RPE‐like behavior. They not only secrete trophic factors that support PR,^14^ but also phagocytose rod outer segments, a key function of the RPE.^13^ In preclinical models of retinal degeneration, subretinally delivered CNS10‐NPC preserve PRs and vision.^13^, ^15^ The formation of a layer of cells that support PR survival suggests that CNS10‐NPC could provide benefit for both AMD and RP patients.

A team led by Shaomei Wang and Clive Svendsen completed GMP manufacturing and IND‐enabling safety studies, refined the surgical delivery technique, and obtained FDA clearance of an IND to proceed with clinical development of CNS10‐NPC in RP. A CIRM‐supported phase 1 trial of CNS10‐NPC in patients with RP is now poised to initiate enrollment (ClinicalTrials.gov Identifier: NCT04284293). It is hoped that allogeneic NPC grafts will persist in the human eye and enable long‐term photoreceptor preservation. The blood retinal barrier (BRB) isolates the retina from circulating leukocytes^16^ and the retina has been hypothesized to be an immune‐privileged site capable of retaining an allogeneic cell graft without eliciting rejection. However, the RP disease process appears to compromise the BRB,^17^, ^18^, ^19^ as evidenced by the frequent formation of macular edema^20^, ^21^; and in addition, subretinal injection breaches the barrier, albeit temporarily. Immunosuppression will therefore be employed in this phase 1 trial.

### 3.4 Alternative approaches for RP

The landscape of regenerative medicine approaches for RP remains limited (Table 2). A number of different allogeneic cell types are under investigation using various routes of delivery and are in early stages of clinical development. The jCyte hRPC approach is entering late stage development having completed a phase 2b trial (as described above).

**TABLE 2:** Summary of clinical stage cell therapy approaches for retinitis pigmentosa

Sponsor	Product	Cell type	Delivery	Phase
jCyte	jCell (hRPC)	Retinal progenitor cells	Intravitreal injection	Phase 2b
Cedars‐Sinai Medical Center	CNS10‐NPC	Neural progenitor cells	Transplantation into subretinal space	Phase 1
ReNeuron	hRPC	Human retinal progenitor cells	Subretinal injection	Phase 1/2a
Jinnah Burn and Reconstructive Surgery Centre, Lahore Pakistan	UMSCs	Umbilical cord derived mesenchymal stem cells	Injection into sub‐tenon or suprachoroidal space	Phase 2
Centre d'Etude des Cellules Souches	ISTEM‐01	Human embryonic stem cell derived RPE on human amniotic membrane	Subretinal implantation	Phase 1/2a

Abbreviations: hRPC, human retinal progenitor cell; NPC, neural progenitor cell; RPE, retinal pigment epithelium; UMSC, umbilical cord stem cell.

## 4 LIMBAL CELL DEFICIENCY

CIRM began funding a LSCD project starting in 2010. Briefly, the clarity and refractive power of the cornea is maintained by limbal stem cells (LSCs) that reside in the limbus, a region located at the edge of the cornea and conjunctiva.^22^ LSCD from chemical injury, contact lens use, or inherited conditions of the eye, disrupts the ability to maintain a clear cornea, resulting in pain, light sensitivity, and vision loss that can lead to blindness. Corneal transplantation is an established therapy for treating corneal damage, but because LSCs are necessary to maintain the transplanted cornea,^22^, ^23^ corneal transplant is not an option for severe LSCD.

An alternative strategy is based on transplantation of ex vivo expanded, autologous LSC from the healthy contralateral eye. Clinical proof of concept for autologous LSC replacement therapy has been demonstrated,^24^, ^25^ and the therapy is currently available in Europe but not in the United States. CIRM grantee Sophie Deng is building on this proven approach with the goal of making LSC replacement therapy available to patients in the United States. The Deng team developed a robust, improved GMP compliant, xenobiotic‐free manufacturing process, conducted preclinical testing of the resultant LSC and secured an active IND. Enrollment of patients in a CIRM funded phase 1 clinical trial of this autologous LSC product (ClinicalTrials.gov Identifier: NCT03957954) is poised to begin.

With CIRM support, Dr Deng addressed an additional roadblock. Understanding LSCD severity is important for selecting a patient's best treatment course. If sufficient LSC remain, an injured cornea may repair itself. With inadequate LSC, an injured cornea can neither repair nor maintain a corneal transplant on its own. Similarly, inadequate LSC in a patient's fellow eye renders it an ineligible donor for autologous transplant. In these cases, either allogeneic transplant with immune suppression or a corneal prosthesis would be indicated. A persistent challenge in the field, however, has been the lack of consistent and quantitative methods to both diagnose and accurately stage LSCD. LSCD diagnosis has relied on clinical symptoms, slit lamp examination, and impression cytology to assess the degree of corneal damage and abnormalities of the corneal surface such as neovascularization or conjunctivalization (as indicated by goblet cells). Unfortunately, reliance solely upon these findings can result in misdiagnosis and selection of an inappropriate therapeutic plan.^26^ Thus, reliable, quantitative LSCD diagnostic and staging tools represented an unmet medical need.

To address this need, Dr Deng and her colleagues in the Cornea Society developed methods and consensus around LSCD diagnosis and staging.^22^, ^27^ The techniques include quantitative in vivo confocal microscopy measures and impression cytology assays. These methods enable identification of residual LSC in patients formerly diagnosed with complete LSCD,^28^, ^29^ suggesting that even patients with clinically complete or bilateral LSCD may be able to benefit from autologous LSC transplant. This may open the possibility of vision restoration without the requirement for immune suppression for these patients.

### 4.1 Alternative approaches for LSCD

The main treatment option for LSCD is a surgical approach using either allogeneic or autologous tissue for the graft. Graft failure due to rejection is a common complication in the case of allografts. Expanded autologous LSC (Holoclar) is approved for use in Europe but not in the United States (as indicated above). A number of alternative autologous approaches are in early stages of clinical development as well as an approach using cadaveric LSC (Table 3).

**TABLE 3:** Summary of clinical stage cell therapy approaches for limbal stem cell deficiency

Sponsor	Product	Cell type	Delivery	Phase
University of California, Los Angeles (UCLA) (Sophie Deng)	cLSC	Autologous limbal stem cells	Transplantation	Phase 1
Holostem Terapie Avanzate s.r.l.	Holoclar	Autologous expanded limbal stem cells	Transplantation	Phase 4
Massachusetts Eye and Ear Infirmary	Cultivated autologous limbal epithelial cell (CALEC) expanded on an amniotic membrane	Autologous limbal epithelial cells	Surgical transplantation	Phase 1/2
RHEACELL GmbH & Co. KG, Germany	LSC2	Cadaveric limbal stem cells	Topical application	Phase 1/2a
CHU de Quebec‐Universite Laval, Canada	Autologous cultured corneal epithelium (CECA)	Autologous corneal epithelium	Transplantation	Phase 1/2
Chang Gung Memorial Hospital, Taipei, Taiwan	COMET	Cultivated autologous oral mucosal epithelial	Transplantation	Phase 1
Hospices Civils de Lyon, France	Autologous jugal mucosa cell sheet (FEMJA)	Cultured autologous oral mucosa epithelial sheet	Transplantation	Phase 1/2

Abbreviation: LSC, limbal stem cell.

## 5 DISCUSSION

Stem cell‐based therapies are showing promise for the treatment of previously intractable debilitating eye diseases. Over the past decade, CIRM funded research focused initially on (a) establishing the feasibility of generating transplantable therapeutic cells from stem cells; (b) assessing the “safe and efficacious” cell dose in preclinical and clinical studies; (c) investigations of cell delivery route; (d) GMP manufacturing of large numbers of cells for preclinical and clinical investigation as well as for potential commercialization; and (e) incorporation of new innovative clinical endpoints in the design of the clinical trials. Later, the first CIRM supported clinical trials in eye diseases demonstrated the feasibility of administering cell therapies to the eye in an outpatient setting, both to the retina as well as to the vitreous. Although longer term data are not yet available and a relatively small number of patients have been treated, there are suggestions that implanted cells survive, are functional, and persist for months with encouraging measurable visual improvement for the patients.

Several regulatory challenges specific to cell‐based therapies needed to be overcome. These included demonstrating that the final cell product does not contain residual undifferentiated, potentially tumorigenic cells and does not induce tumors in preclinical animal models. Additional challenges included developing a potency assay and demonstrating manufactured lot consistency when the cellular product's mechanism of action is complex and not well understood. In some cases, a specifically designed delivery tool needed to be developed and tested in large animal preclinical models.

The optimal route of delivery of a cell therapy for a retinal disease will likely depend on the mechanism of action. For a therapy that exerts a neuroprotective effect via a paracrine mechanism, intravitreal delivery appears to be the preferred route, allowing for diffusible factors to broadly access the retina. Intravitreal delivery has several additional advantages in that it is minimally invasive, allows for repeat injection and is routinely used in outpatient settings, including in the optometrist's office. In contrast, retinal cell replacement strategies may necessitate subretinal delivery, requiring specific tools and surgical expertise that may best be deployed at centers of excellence. A CIRM supported study has demonstrated that subretinal delivery is feasible and that durable graft retention can be achieved. The potential for long‐term clinical benefit thus could outweigh the risks.

The need for immunosuppression remains an open question. Whether the eye is an immune privileged site remains to be determined through continued clinical investigation and immune monitoring. Nevertheless, surgical delivery of cells to the retina may temporarily breach the blood‐retinal barrier. To guard against possible immune rejection of transplanted cells, current clinical trial protocols include immunosuppression for a period of time. Important questions that remain to be resolved include how much immunosuppression is required and for how long, and whether systemic immunosuppression can be replaced with local immunosuppression.

### 5.1 Future directions

The development of stem cell therapies for debilitating eye diseases progressed rapidly and measurably on several fronts during the past 5 years. Advancements include (a) the development of innovative tools and technologies that incorporate patients' QOL measures; (b) evolution of a regulatory paradigm that previously required a change of 13 letters on an eye chart in order to approve a therapy to acceptance of a mobility endpoint when appropriate; and (c) the advancement of gene therapy approaches for some rare eye diseases. New therapeutic modalities and genomics will expand the field further.

As with all cell therapies, the development of a robust and scalable manufacturing process is critical to the final success of the therapy. The increasing adoption of expedited regulatory pathways, including regenerative medicine advanced therapy designation, has escalated the importance of defining critical quality attributes for each cell and gene product as early in the process as possible and of rapidly establishing a mature manufacturing process suitable for commercialization.

## CONFLICT OF INTEREST

The authors declared no potential conflicts of interest.

## AUTHOR CONTRIBUTION

All authors contributed significantly to the conception, design, writing, and review of the manuscript.

## Data Availability

Data sharing not applicable to this article as no data sets were generated or analyzed during the current study.
